# Symptoms and signs of long COVID: A rapid review and meta-analysis

**DOI:** 10.7189/jogh.12.05014

**Published:** 2022-05-21

**Authors:** Quin Healey, Aziz Sheikh, Luke Daines, Eleftheria Vasileiou

**Affiliations:** 1 Edinburgh Medical School, The University of Edinburgh, Edinburgh, UK; 2 Usher Institute, The University of Edinburgh, Edinburgh, UK

## Abstract

**Background:**

Long COVID is defined as symptoms and signs related to severe acute respiratory syndrome coronavirus 2 (SARS-CoV-2) that are present at least four weeks following acute infection. These symptoms and signs are poorly characterised but may be associated with significant morbidity. We sought to synthesise the evidence on their incidence to guide future research, policy and practice.

**Methods:**

We searched Medline and Embase for longitudinal cohort studies from January 2020 to July 2021 that investigated adults with long COVID at least four weeks after acute infection. Risk of bias was assessed using the Joanna Briggs Institute checklist for cohort studies. Random-effects meta-analyses were performed with subgroup analysis by follow-up time (4-12 vs more than 12 weeks).

**Results:**

19 studies were included, 13 of which included patients hospitalised with COVID-19. The total sample size was 10 643 and the follow-up time ranged from 30 to 340 days. Risk of bias was assessed as high in one study, moderate in two studies and low in the remaining 16 studies. The most common symptoms and signs seen at any time point in long COVID were fatigue (37%; 95% confidence interval (CI) = 23-55), dyspnoea (21%; 95% CI = 14-30), olfactory dysfunction (17%; 95% CI = 9-29), myalgia (12%; 95% CI = 5-25), cough (11%; 95% CI = 6-20) and gustatory dysfunction (10%; 95% CI = 7-17). High heterogeneity was seen for all meta-analyses and the presence of some funnel plot asymmetry may indicate reporting bias. No effect of follow-up time was found for any symptom or sign included in the subgroup analysis.

**Conclusions:**

We have summarised evidence from longitudinal cohort studies on the most common symptoms and signs associated with long COVID. High heterogeneity seen in the meta-analysis means pooled incidence estimates should be interpreted with caution. This heterogeneity may be attributable to studies including patients from different health care settings and countries.

Long COVID is defined as symptoms and signs related to severe acute respiratory syndrome coronavirus 2 (SARS-CoV-2) that are present at least four weeks following acute infection [1]. It can be further described as either ongoing symptomatic COVID-19, from 4-12 weeks, or post-COVID-19 syndrome, from 12 weeks onward [1]. Symptoms and signs of long COVID are poorly characterised and may be associated with significant morbidity [2].

SARS-CoV-2 began to circulate in December 2019, was declared a pandemic in March 2020 [3], and has since infected over 300 million individuals globally [4]. Long COVID continues to become an increasing issue over time, resulting in considerable morbidity and mounting costs to health services [5]. Because it is such a rapidly evolving field, rapid systematic reviews are important for maintaining an informed understanding of the condition. This might allow for more effective diagnosis and guide both policy decisions and future research.

We wanted to identify the incidence of symptoms and signs of long COVID and to investigate if they differed in patients with ongoing symptomatic COVID-19 and post-COVID-19 syndrome. This review differs from others [6-8] as it only includes longitudinal cohort studies with data on symptoms and signs from the acute infection. By confirming that identified symptoms were also present at this time point, it increases the likelihood that they are related to SARS-CoV-2 infection rather than to comorbidities. Misclassification bias is therefore reduced compared to using cross-sectional studies. This review also includes more recently published studies, which allow for longer follow-up times and may start to include vaccinated individuals.

## METHODS

A protocol was developed prior to conducting the review based on preferred reporting items for systematic review and meta-analysis protocols (PRISMA-P) guidelines [9] (Appendix S1 in the **Online Supplementary Document**).

### Eligibility criteria

Eligibility criteria for study inclusion were: longitudinal cohort studies; adults with long COVID, defined as symptoms and signs related to SARS-CoV-2 present at least four weeks after acute infection; studies reported from January 2020 to July 2021; studies available in English so their relevance and contents could be confirmed by a fluent speaker; and data available from the acute infection.

### Search methods

We used two databases: Medline and Embase. The search strategy involved medical subject headings and text words related to symptoms and signs of long COVID and is available in Appendix S2 in the **Online Supplementary Document**. Backward searching for additional studies was not carried out, following recommendations by the Cochrane Rapid Reviews Methods Group [10].

### Study screening

The screening was carried out by a single reviewer (QH) and guided by the PRISMA flow diagram. Potential eligibility was initially assessed based on the title and abstract. Full-text articles were screened to confirm which studies met the inclusion criteria. An additional reviewer (EV) provided guidance when eligibility was unclear and assessed all the included studies to ensure eligibility.

### Data extraction

Data were extracted from each study by a single reviewer (QH), using a data extraction form designed for this review (Table S1 in the **Online Supplementary Document**). It included study design, country, follow-up time, results, and demographic information. The collected demographic data included age, health care setting, comorbidities, ethnicity, and sex. Follow-up time was converted into days to allow comparison between studies, with the assumption that a month equalled 30.4 days.

### Risk of bias assessment

Two reviewers (QH, EV) assessed the risk of bias in each study using the Joanna Briggs Institute checklist for cohort studies [11]. This list contains 11 components relating to factors like exposure measurement, confounders, outcome measurement, follow-up, and statistics. Each component was assessed as “yes”, “no”, “unclear” or “not applicable”. Surveys of patient-reported symptoms were deemed reliable measures of outcomes, as there was often no alternative. Patient recollection of previous symptoms was deemed unreliable due to the risk of recall bias [12]. In line with other systematic reviews [13-15], the overall risk of bias for each study was based on the proportion of components that were answered “yes”. Studies were judged as having low (>70% of components = “yes”), moderate (50%-70% of components = “yes”), or high (<50% of components = “yes”) risk of bias.

### Data analysis

Meta-analyses were performed using the meta package in RStudio (V.1.3.959). Pooled incidence and 95% confidence intervals (CI) were calculated for symptoms and signs reported in at least five studies. The rationale for this was to focus on the most important symptoms and signs rather than rarely reported, potentially incidental findings highlighted in similar reviews [6-8]. The reporting of olfactory-gustatory dysfunction proportion was used when present for calculating the pooled incidence of both olfactory dysfunction and gustatory dysfunction. One study reported the proportion of “diarrhoea or vomiting” [16]. This was used when calculating the pooled incidence of diarrhoea, because the other included studies that assessed vomiting found that no subjects experienced it [17,18]. Heterogeneity was assessed using *I*^2^ statistics, with values of 25%, 50%, and 75% representing low, moderate, and high heterogeneity, respectively. Due to differing study populations and the heterogeneity found, random-effects meta-analyses were undertaken. Subgroup analysis was carried out by follow-up time: 4-12 vs >12 weeks. Funnel plots were produced by Egger’s method, with the logit transformed proportion against standard error. Binary outcomes were presented as percentages of patients, while continuous outcomes were presented as mean or median throughout.

## RESULTS

### Study selection

**Figure 1:**
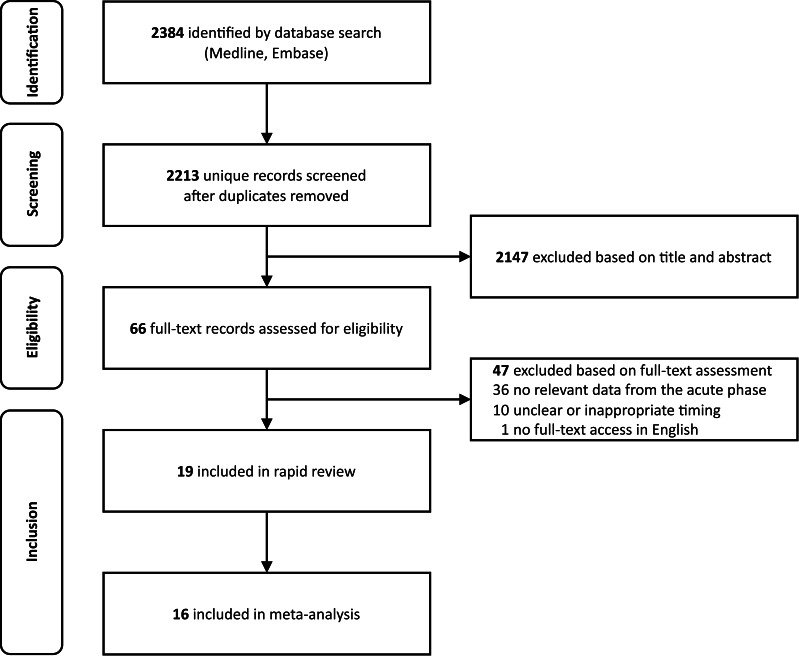
PRISMA flowchart of the study selection process.

2384 studies were identified during the initial database search and 2213 unique records remained after deduplication. The title and abstract of each study were screened for eligibility, followed by the full text if the study passed the initial eligibility assessment. 19 of the 66 studies met the inclusion criteria and 16 were included in the meta-analysis. The remaining three studies were excluded from the meta-analysis as their data were presented graphically, without accompanying raw data. **Figure 1** shows a PRISMA flowchart of the study selection process.

### Study characteristics

**Table 1** summarises the demographic data, timing, and results of the 19 included cohort studies. The median study sample size was 145 and the total sample size was 10 643. Studies were carried out across the world: 79% in Europe, 11% in Asia, 5% in North America and 5% in Australia. They also included participants from different health care settings: 42% only hospitalised, 21% only non-hospitalised, 26% both hospitalised and non-hospitalised and 11% not stated. Average participant age was 35-64 years and a median 47% were female. Commonly stated comorbidities included obesity, hypertension, diabetes, respiratory disease, and ischaemic heart disease. Follow-up time ranged from 30 to 340 days. Only 26% of studies reported participant ethnicity, so it was not possible to explore if it affected patient outcomes.

**Table 1:** Characteristics of the included studies

Author (country)	Hospital (%) {ICU (%)}	Age (years)	Comorbidities	Follow-up time (days)	Body system	Results
Bellan (Italy) [19]	100 {12}	61	41% hypertension, 15% diabetes, 11% obesity, 11% endocrine disease, 10% malignancy, 9% IHD, 8% dyslipidaemia, 7% AF, 6% COPD, 6% CKD, 6% haematological disease, 5% anxiety/depression, 4% cerebrovascular disease, 3% liver disease, 3% VTE, 2% IBD, 2% autoimmune disease	107	Generalised/MSK	5.9% myalgia, 5.9% arthralgia
					Respiratory	5.5% dyspnoea, 2.5% cough, 0.4% chest pain, 51.6% reduced DLCO, normal spirometry
					Neuropsychiatric	43% PTSD symptoms
					ENT	5% gustatory dysfunction, 4.6% olfactory dysfunction
					Gastrointestinal	1.3% diarrhoea
Bliddal (Denmark) [20]	0	50	28% allergy, 17% osteoarthritis, 15% hypertension, 9% thyroid disease, 8% asthma	84	Generalised/MSK	Fatigue, myalgia, arthralgia, chills, fever
					Respiratory	Dyspnoea, cough, chest pain, sputum production
					Neuropsychiatric	Memory issues, concentration issues, headache
					ENT	Olfactory dysfunction, gustatory dysfunction, sore throat, rhinorrhoea, sneezing
					Gastrointestinal	Diarrhoea, anorexia, abdominal pain, nausea
					Others	Red runny eyes
Chiesa-Estomba (Italy) [21]	Not stated	41	6% hypertension, 6% hypothyroidism, 6% asthma, 4% autoimmune disease, 3% diabetes, 2% IHD, 1% COPD	47	ENT	51% olfactory dysfunction
Cousyn (France) [22]	0	35	Not stated	60	ENT	16.8% olfactory dysfunction, 9.6% gustatory dysfunction
Daher (Germany) [17]	100	64	59% hypertension, 25% diabetes, 22% CKD, 19% IHD, 13% asthma, 9% COPD, 9% AF, 9% heart failure	56	Generalised/MSK	45% fatigue, 15% myalgia, 3% fever, slight pain/discomfort
					Respiratory	33% dyspnoea, 33% cough. Normal spirometry, normal ABG, reduced DLCO, reduced distance on 6MWT
					Neuropsychiatric	18% cognitive issues, 15% headache, mild depression, subthreshold anxiety
					ENT	12% olfactory dysfunction, 12% rhinorrhoea, 9% gustatory dysfunction, 9% sore throat
					Gastrointestinal	9% diarrhoea, 6% nausea, 3% abdominal pain, normal LFTs
					Cardiovascular	18% angina, normal left ventricular function, normal right ventricular function, normal cardiac biomarkers
					Other biomarkers	Normal FBC, normal coagulation screen, raised ferritin, potentially raised D-dimer, normal U&Es, normal CRP, normal procalcitonin, normal TFTs, normal IL-6
Fernandez-de-Las-Penas (Spain) [23]	100 {7}	61	26% hypertension, 12% diabetes, 12% IHD, 7% asthma, 5% obesity, 4% COPD, 2% cerebrovascular disease, 2% rheumatological disease	340	Generalised/MSK	61.2% fatigue
					Respiratory	23.3% dyspnoea, 6.5% chest pain, 2.5% cough
Froidure (Belgium) [24]	100 {22}	60	47% hypertension, 42% dyslipidaemia, 28% obesity, 22% diabetes, 9% asthma, 4% COPD, 2% lung cancer, 1% ILD	98	Generalised/MSK	25% fatigue
					Respiratory	Abnormal chest CT: 67% ground glass opacities, 44% reticulations, 20% fibrotic lesions/traction bronchiectasis, 7% consolidations. 46% reduced DLCO, 35% dyspnoea, 10% dry cough, 4% chest tightness, normal spirometry
Gerhards (Germany) [25]	10	46	Not stated	183	Generalised/MSK	17% fatigue
					Neuropsychiatric	Depression, concentration issues
					ENT	27% olfactory/gustatory dysfunction
					Others	Alopecia
Ghosn (France) [26]	100 {29}	61	38% hypertension, 22% obesity, 19% diabetes, 18% IHD, 10% COPD, 7% CKD, 7% malignancy, 1% liver disease	194	Generalised/MSK	Fatigue, arthralgia, myalgia
					Respiratory	Dyspnoea, cough
					Neuropsychiatric	Headache
					ENT	Rhinorrhoea, olfactory dysfunction, gustatory dysfunction, sore throat
Han (China) [27]	100	54	28% hypertension, 14% respiratory disease, 11% diabetes	175	Respiratory	62% abnormal chest CT: 35% fibrotic-like changes, 27% ground glass opacities/interstitial thickening, nodules/masses, interlobar pleural traction, pulmonary atelectasis and bronchiectasis. 26% reduced DLCO, 14% mild dyspnoea, 10% sputum production, 6.1% dry cough
Holmes (Australia) [28]	0	57	Not stated	183	Generalised/MSK	50% fatigue, 35.7% arthralgia, 21.4% myalgia
					Respiratory	28.6% cough, 25% dyspnoea, 3.6% chest pain
					Neuropsychiatric	10.7% headache
					ENT	28.6% olfactory dysfunction, 14.3% rhinorrhoea
					Gastrointestinal	No abdominal pain
Jacobs (USA) [29]	100	57	49% obesity, 48% hypertension, 28% diabetes, 12% IHD, 11% dyslipidaemia, 10% asthma, 10% malignancy, 5% arrhythmia, 4% COPD, 4% hypothyroidism, 4% depression, anxiety or schizophrenia, 3% heart failure, 3% sleep apnoea, 2% VTE	35	Generalised/MSK	44.8% fatigue, 21.3% myalgia, 15.8% arthralgia, 1.1% fever, 1.1% ulcer
					Respiratory	31.7% dyspnoea, 25.1% cough, 14.8% sputum production
					Neuropsychiatric	12.6% headache, 8.7% cognitive issues
					ENT	9.8% gustatory dysfunction, 9.3% olfactory dysfunction
					Gastrointestinal	3.8% diarrhoea
					Others	8.2% eye irritation, 1.1% ulcer
Leth (Denmark) [30]	100 {12}	58	36% obesity, 29% hypertension, 12% malignancy, 10% IHD, 8% asthma, 8% COPD, 4% diabetes, 4% hyperthyroidism, 2% cerebrovascular disease	128	Generalised/MSK	63% fatigue, 35% myalgia
					Respiratory	53% dyspnoea, 24% cough, 20% chest pain, 12% sputum production
					Neuropsychiatric	45% concentration issues, 27% headache, 27% paraesthesia
					ENT	31% gustatory dysfunction, 27% olfactory dysfunction, 10% sore throat
					Gastrointestinal	10% abdominal pain, 8% diarrhoea, 8% nausea, 4% anorexia
Mahmud (Bangladesh) [18]	Not stated	40	15% hypertension, 14% diabetes	30	Generalised/MSK	33% fatigue, 1.4% arthralgia, 0.6% myalgia
					Respiratory	8.5% cough, 7% dyspnoea, 0.8% chest pain
					Neuropsychiatric	3.9% circadian rhythm disorders, 3.4% headache, 2.3% sleep disturbance, 1.4% adjustment disorder
					ENT	2.3% vertigo, 2% olfactory dysfunction
					Cardiovascular	1.4% palpitation
Otte (Germany) [31]	0	45	Not stated	201	ENT	42.3% subjective olfactory dysfunction, 26.9% objective olfactory dysfunction (discrimination and identification issues)
Peghin (Italy) [32]	26	53	23% hypertension, 16% obesity, 6% diabetes, 4% respiratory disease, 1% IHD, 2% liver disease, 1% depression/anxiety, 0% CKD	191	Generalised/MSK	13.1% fatigue, 8.2% rheumatological issues
					Respiratory	6% dyspnoea, 2% cough, 0.8% chest pain
					Neuropsychiatric	9.6% neurological disorders, 4.9% psychiatric disorders, 2.7% headache
					ENT	10.4% olfactory/gustatory dysfunction,
					Gastrointestinal	1.5% gastrointestinal disorders
					Others	3.7% alopecia, 3.4% cutaneous manifestations, 0.3% ocular symptoms
Sonnweber (Austria) [16]	75	57	40% cardiovascular disease, 30% hypertension, 19% dyslipidaemia, 17% diabetes, 7% asthma, 7% CKD, 6% COPD, 6% liver disease, 6% malignancy, 1% ILD	103	Generalised/MSK	24% night sweats, 0% fever
					Respiratory	63% abnormal chest CT: ground-glass opacities, reticular lesions, consolidations, bronchial dilation. 36% dyspnoea, abnormal spirometry: 22% reduced FVC, 22% reduced FEV1, normal FEV1/FVC. 21% reduced DLCO, 17% cough
					Neuropsychiatric	22% sleep disorders
					ENT	19% olfactory dysfunction
					Gastrointestinal	9% diarrhoea/vomiting
					Cardiovascular	97% normal LVEF, 55% diastolic dysfunction on echo, 23% raised NT-proBNP, 10% pulmonary hypertension, 1% pericardial effusion
					Other biomarkers	Raised D-dimer, potentially raised ferritin, normal CRP, normal procalcitonin, normal IL-6
Sudre (UK, USA, Sweden) [33]	14	42	26% obesity, 14% respiratory disease, 10% asthma, 3% diabetes, 2% IHD, 1% CKD	84	Generalised/MSK	Fatigue, myalgia, fever
					Respiratory	Dyspnoea, cough, chest pain
					Neuropsychiatric	Headache, paraesthesia, numbness, concentration/ memory issues
					ENT	Olfactory dysfunction, sore throat, hoarse voice, tinnitus, earache
					Gastrointestinal	Diarrhoea, abdominal pain
					Cardiovascular	Palpitations/tachycardia
Vaira (Italy) [34]	23	51	29% obesity, 27% IHD, 15% respiratory disease, 11% diabetes	60	ENT	21% olfactory dysfunction, 7.9% gustatory dysfunction

ICU – intensive care unit, IHD – ischaemic heart disease, AF – atrial fibrillation, COPD – chronic obstructive pulmonary disease, CKD – chronic kidney disease, VTE – venous thromboembolism, IBD – inflammatory bowel disease, NS – not stated, ILD – interstitial lung disease, MSK – musculoskeletal, ENT – ear, nose, and throat, OGD – olfactory-gustatory dysfunction, DLCO – diffusing capacity for carbon monoxide, PTSD – posttraumatic stress disorder, ABG – arterial blood gas, 6MWT – 6-min walk test, LFT – liver function test, FBC – full blood count, U&E – urea and electrolyte, CRP – c-reactive protein, TFT – thyroid function test, IL-6 – interleukin-6, FVC – forced vital capacity, FEV1 – forced expiratory volume in one second, NT-proBNP – N-terminal pro B-type natriuretic peptide

### Risk of bias

One study was assessed as having high risk of bias, two as having moderate risk of bias, and 16 as having low risk of bias. The reasoning for these assessments is summarised in Table S2 in the **Online Supplementary Document**. A key issue was that any symptoms and signs prior to infection possibly related to comorbidities were rarely reported. It was unclear whether identified symptoms and signs could be attributed to SARS-CoV-2 infection. Other issues included not dealing with cofounding factors like comorbidities or not measuring the outcomes in a valid and reliable way.

### Meta-analysis

**Figure 2:**
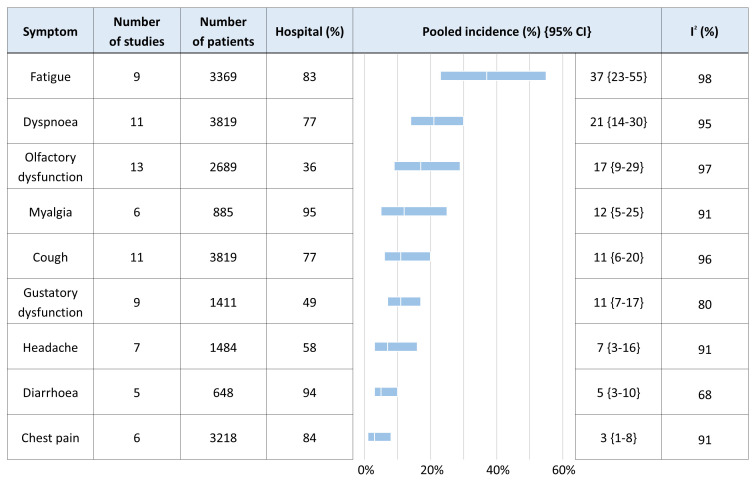
Meta-analysis of symptoms reported by at least five studies.

Forest plots for each symptom reported by at least five studies are presented in Figure S1 in the **Online Supplementary Document** and the pooled incidence from each meta-analysis is outlined in **Figure 2**. The most common symptoms and signs seen at any time point in long COVID were fatigue (37%; 95% CI = 23-55), dyspnoea (21%; 95% CI = 14-30), olfactory dysfunction (17%; 95% CI = 9-29), myalgia (12%; 95% CI = 5-25), cough (11%; 95% CI = 6-20) and gustatory dysfunction (10%; 95% CI = 7-17). Less common symptoms were headache (7%; 95% CI = 3-16), diarrhoea (5%; 95% CI = 3-10) and chest pain (3%; 95% CI = 1-8). The percentage of hospitalised patients in each meta-analysis ranged from 36% to 94%. High heterogeneity was seen, ranging from 68% to 98%. Subgroup analysis by follow-up time is summarised in Table S3 in the **Online Supplementary Document**. No significant difference in incidence was found between 4-12 and >12 weeks for any symptom, and heterogeneity remained high in most symptoms. Funnel plots for each symptom are presented in Figure S2 in the **Online Supplementary Document**. The plots for fatigue, olfactory dysfunction, and chest pain appear asymmetrical, but it was not possible to assess this statistically due to the small number of included studies [35].

## DISCUSSION

### Summary of findings

This review found that the most common symptoms and signs of long COVID were fatigue (37%), dyspnoea (21%), olfactory dysfunction (17%), myalgia (12%), cough (11%), and gustatory dysfunction (11%). Pooled incidence estimates did not differ between ongoing symptomatic COVID-19 and post-COVID-19 syndrome.

### Strengths and limitations

This review followed PRISMA guidelines, ensuring rigorous methods. However, most stages were carried out by a single reviewer, increasing the risk of inappropriate screening [36] or improper data extraction [37]. Our inclusion criteria required studies to have acute-phase data, to increase the likelihood that symptoms were related to the virus. This may be important as some studies had very large follow-up times or a high proportion of participants with comorbidities. However, this criterion excluded some potentially useful studies, such as those that only used measures that were unlikely to be used in the acute phase. Examples include cardiac magnetic resonance imaging (MRI) [38-40] and cognitive assessment [41], and there could be an underrepresentation of issues assessed via these investigations. We only included studies available in English, which may have affected the geographical distribution of included studies. As no included studies were carried out in Africa or South America, and only 11% of studies were carried out in Asia, this review might not adequately capture the regional variations of long COVID.

We appraised the included studies using a well-validated tool and found they generally had low risk of bias. However, confounding by severity may be present due to patient recruitment during hospital admission or attendance in outpatient departments and may lead to overestimation in our pooled incidence data. For example, all patients in three studies [21,22,31] had developed olfactory-gustatory dysfunction in the acute phase, compared to an estimated 47% of the general population [42]. Furthermore, misclassification bias may be present, as identified symptoms may be caused by comorbidities or superinfection. Comorbidities could be resolved in future studies by including COVID-negative controls. Superinfection could be mitigated by measuring inflammatory markers, such as interleukin-6 and C-reactive protein, which are elevated in acute infection [43,44] but not in long COVID [16,17]. High heterogeneity in the meta-analysis (median = 91%) means that pooled incidence estimates should be interpreted with caution. Without understanding the heterogeneity’s source, these estimates may poorly represent outcomes for individuals. Possible sources include studies with patients from different health care settings and countries. The heterogeneity is less likely to be explained by follow-up time as it remained high after subgroup analysis. Finally, funnel plot asymmetry may reflect reporting bias.

### Comparison with existing literature

Our findings correspond with those of similar reviews, such as Martimbianco et al. [6], Michelen et al. [7], and Lopez-Leon et al. [8]. The latter two carried out meta-analyses that reported the same common symptoms identified in this review, such as fatigue and dyspnoea. This is particularly encouraging because different inclusion criteria resulted in little overlap of included studies. We report very similar symptom rates to Michelen et al. [7], but much lower rates than Lopez-Leon et al. [8]. The latter may be explained by their inclusion of subjects assessed less than four weeks after SARS-CoV-2 infection or our inclusion of more recent studies that are likely to involve at least some vaccinated subjects and a greater proportion of individuals with natural immunity following infection. There may also have been an impact from different variants circulating at different time points. Our review did not report weakness or malaise although both were found to be common based on a small number of studies in Michelen et al. [7]. These differences likely relate to our different inclusion criteria and indicate a need for further high-quality primary research to investigate these symptoms.

### Implications

Identification of the most common long COVID symptoms may influence future research and policy. These symptoms could benefit the most from research to understand their pathogeneses and trial treatments. They may also benefit from an increased focus on long COVID clinical pathways, such as increased education of health care workers in long COVID clinics. Furthermore, the identification of limitations in the studies included in this review, such as confounding by severity and misclassification bias, may guide the methodology of future research.

## CONCLUSION

This rapid review identified the most common symptoms and signs of long COVID: fatigue, dyspnoea, olfactory dysfunction, myalgia, and cough. The included studies were found to be at an overall low risk of bias, but a high level of heterogeneity arose in the meta-analyses. This may indicate an effect of different study populations on the results. Further primary research is required to confirm the characteristics of long COVID, understand its pathogeneses, and propose treatments.

## Additional material


Online Supplementary Document
