# Supplementary material for: Symptoms and signs of long COVID: A rapid review and meta-analysis
Source: J Glob Health. 2022 May 21;12:05014. doi: 10.7189/jogh.12.05014 (PMC9125197; doi:10.7189/jogh.12.05014)
Supplement: Online Supplementary Document [file jogh-12-05014-s001.pdf]

Supplementary Material

Appendix S1 Review protocol

We will conduct a rapid literature review of published and unpublished reports. The latter may be important as long COVID is such a rapidly evolving topic. Reports will be selected according to the criteria outlined below.

Study design

Observational studies will be eligible for inclusion while systematic reviews and meta-analyses will only be used for identifying additional studies.

Participants

We will include papers that examine adults with long COVID. This is defined as signs or symptoms related to SARS-CoV-2 that are present 4 weeks following suspected or confirmed acute infection.

Timing

Studies reported between January 2020 to July 2021 will be included in the review. This start date has been chosen because it appears to be the earliest date of publish of a primary long COVID study.

Language

Only studies reported in English will be included so their relevance and contents can be confirmed by a fluent speaker.

Search Methods

Literature searching will involve medical subject headings (MeSH) and text words related to signs and symptoms of long COVID. We will search four databases: Medline, Embase, MedRxiv and BioRxiv. We will use the search strategy:

1. COVID-19/ or SARS-CoV-2/
2. (covid* or SARS-CoV-2).tw.
3. or/1-2
4. (long* or chronic or post*).tw.
5. Signs and symptoms/
6. (sign* or symptom* or present* or epidemiolog* or complication* or impair* or consequence*).tw.
7. or/5-6
8. 3 and 4 and 7

Study titles and abstracts will be screened for potential eligibility followed by full text screening to confirm they meet the inclusion criteria. When eligibility is unclear, an additional reviewer will provide guidance.

Risk of Bias Assessment

A single reviewer will assess the risk of bias of each study using the Joanna Briggs Institute (JBI) checklist for cross-sectional studies. This contains 8 components and results in the decision to either include or exclude the study.

Data Extraction

Data will be extracted from each study by a single reviewer, using a form designed for this review. It will include demographic information, study type, methodology, results and extra useful information.

Data Analysis

If enough compatible data are available, meta-analysis will be performed to estimate the prevalence of the signs and symptoms of long COVID. All statistical analyses will be undertaken using R.

Appendix S2 Search strategy

1. COVID-19/ or SARS-CoV-2/
2. (covid* or SARS-CoV-2).tw.
3. or/1-2
4. (long* or chronic or post*).tw.
5. Signs and symptoms/
6. (sign* or symptom* or present* or epidemiolog* or complication* or impair* or consequence*).tw.
7. or/5-6
8. 3 and 4 and 7

Table S1 Data extraction form

Author	Country	Hospitalisation % (ICU %)	Age	Comorbidities	Ethnicity

Timing (days)	Timing description (after symptom onset/ diagnosis/ discharge	Sample size	Generalised and MSK	Biomarkers	Respiratory

Neuropsychiatric	Sensory specialities	Gastrointestinal	Cardiovascular	Others	

Table S2 Risk of bias assessment

Author	1	2	3	4	5	6	7	8	9	10	11	Overall
Bellan	NA	NA	✓	✓	✓	U	✓	✓	NA	NA	✓	Low
Bliddal	NA	NA	✓	✓	✓	U	✓	✓	NA	NA	✓	Low
Chiesa-Estomba	NA	NA	✓	✓	✓	U	✓	✓	✓	X	✓	Low
Cousyn	NA	NA	✓	✓	✓	U	✓	✓	✓	U	✓	Low
Daher	NA	NA	✓	✓	X	U	✓	✓	NA	NA	✓	Low
Fernandez-de-Las-Penas	NA	NA	✓	✓	✓	U	✓	✓	NA	NA	✓	Low
Froidure	NA	NA	✓	✓	✓	U	✓	✓	NA	NA	✓	Low
Gerhards	NA	NA	✓	✓	X	U	✓	✓	X	U	✓	Moderate
Ghosn	NA	NA	✓	✓	✓	U	✓	✓	✓	✓	✓	Low
Han	NA	NA	✓	✓	✓	U	✓	✓	NA	NA	✓	Low
Holmes	NA	NA	U	X	X	U	U	✓	NA	NA	✓	High
Jacobs	NA	NA	✓	✓	X	U	X	✓	NA	NA	✓	Moderate
Leth	NA	NA	✓	✓	✓	U	✓	✓	✓	NA	✓	Low
Mahmud	NA	NA	✓	✓	✓	U	✓	✓	✓	NA	✓	Low
Otte	NA	NA	✓	X	X	✓	✓	✓	✓	NA	✓	Low
Peghin	NA	NA	✓	✓	✓	U	U	✓	NA	NA	✓	Low
Sonnweber	NA	NA	✓	✓	✓	U	✓	✓	✓	NA	✓	Low
Sudre	✓	✓	✓	✓	✓	✓	✓	✓	U	U	✓	Low
Vaira	NA	NA	✓	✓	✓	U	✓	✓	✓	NA	✓	Low

Abbreviations: ✓, yes; x, no; U, unclear; NA, not applicable.

Figure S1 Individual forest plots for symptoms and signs included in the meta-analysis

Symptom	Forest plot

Fatigue

Study	Events	Total	Proportion	95%-CI	Weight
Daher	15	33	0.45	[0.28; 0.64]	10.6%
Fernandez-de-Las-Penas	1193	1950	0.61	[0.59; 0.63]	11.7%
Froidure	32	126	0.25	[0.18; 0.34]	11.3%
Gerhards	8	47	0.17	[0.08; 0.31]	10.3%
Holmes	14	27	0.52	[0.32; 0.71]	10.3%
Jacobs	82	183	0.45	[0.37; 0.52]	11.5%
Leth	31	49	0.63	[0.48; 0.77]	10.9%
Mahmud	117	355	0.33	[0.28; 0.38]	11.6%
Peghin	78	599	0.13	[0.10; 0.16]	11.6%
**Random effects model**		**3369**	**0.37**	**[0.23; 0.55]**	**100.0%**
Heterogeneity: $I^2 = 98\%$, $p < 0.01$					

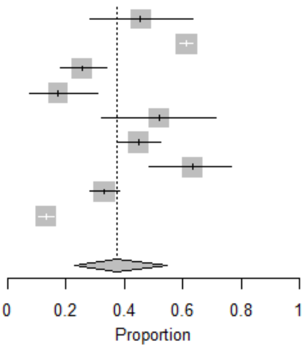

Dyspnoea

Study	Events	Total	Proportion	95%-CI	Weight
Bellan	13	238	0.05	[0.03; 0.09]	8.8%
Daher	11	33	0.33	[0.18; 0.52]	8.2%
Fernandez-de-Las-Penas	454	1950	0.23	[0.21; 0.25]	10.0%
Froidure	44	126	0.35	[0.27; 0.44]	9.5%
Han	16	114	0.14	[0.08; 0.22]	9.0%
Holmes	7	27	0.26	[0.11; 0.46]	7.6%
Jacobs	58	183	0.32	[0.25; 0.39]	9.6%
Leth	26	49	0.53	[0.38; 0.67]	8.8%
Mahmud	25	355	0.07	[0.05; 0.10]	9.4%
Peghin	36	599	0.06	[0.04; 0.08]	9.6%
Sonnweber	52	145	0.36	[0.28; 0.44]	9.5%
**Random effects model**		**3819**	**0.21**	**[0.14; 0.30]**	**100.0%**
Heterogeneity: $I^2 = 95\%$, $p < 0.01$					

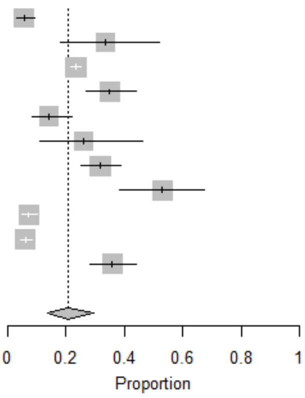

Olfactory dysfunction

Study	Events	Total	Proportion	95%-CI	Weight
Bellan	11	238	0.05	[0.02; 0.08]	7.7%
Chiesa-Estomba	383	751	0.51	[0.47; 0.55]	8.2%
Cousyn	16	98	0.16	[0.10; 0.25]	7.8%
Daher	4	33	0.12	[0.03; 0.28]	6.9%
Gerhards	13	47	0.28	[0.16; 0.43]	7.7%
Holmes	8	27	0.30	[0.14; 0.50]	7.3%
Jacobs	17	183	0.09	[0.06; 0.14]	7.9%
Leth	13	49	0.27	[0.15; 0.41]	7.7%
Mahmud	7	355	0.02	[0.01; 0.04]	7.5%
Otte	11	26	0.42	[0.23; 0.63]	7.4%
Peghin	62	599	0.10	[0.08; 0.13]	8.1%
Sonnweber	28	145	0.19	[0.13; 0.27]	8.0%
Vaira	29	138	0.21	[0.15; 0.29]	8.0%
**Random effects model**		**2689**	**0.17**	**[0.09; 0.29]**	**100.0%**
Heterogeneity: $I^2 = 97\%$, $p < 0.01$					

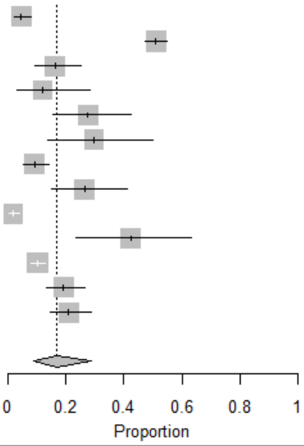

Myalgia

Study	Events	Total	Proportion	95%-CI	Weight
Bellan	14	238	0.06	[0.03; 0.10]	18.0%
Daher	5	33	0.15	[0.05; 0.32]	15.9%
Holmes	6	27	0.22	[0.09; 0.42]	16.2%
Jakobs	39	183	0.21	[0.16; 0.28]	18.7%
Leth	17	49	0.35	[0.22; 0.50]	17.8%
Mahmud	2	355	0.01	[0.00; 0.02]	13.3%
**Random effects model**		**885**	**0.12**	**[0.05; 0.25]**	**100.0%**
Heterogeneity: $I^2 = 91\%$, $p < 0.01$					

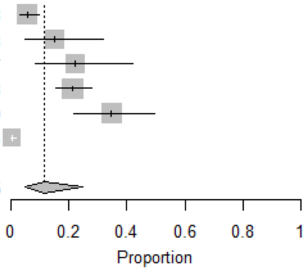

Cough

Study	Events	Total	Proportion	95%-CI	Weight
Bellan	6	238	0.03	[0.01; 0.05]	8.6%
Daher	11	33	0.33	[0.18; 0.52]	8.8%
Fernandez-de-Las-Penas	49	1950	0.03	[0.02; 0.03]	9.5%
Froidure	13	126	0.10	[0.06; 0.17]	9.1%
Han	18	114	0.16	[0.10; 0.24]	9.2%
Holmes	8	27	0.30	[0.14; 0.50]	8.5%
Jacobs	46	183	0.25	[0.19; 0.32]	9.5%
Leth	12	49	0.24	[0.13; 0.39]	8.9%
Mahmud	30	355	0.08	[0.06; 0.12]	9.4%
Peghin	12	599	0.02	[0.01; 0.03]	9.1%
Sonnweber	25	145	0.17	[0.11; 0.24]	9.3%
**Random effects model**		**3819**	**0.11**	**[0.06; 0.20]**	**100.0%**

Heterogeneity: $I^2 = 96\%$, $p < 0.01$

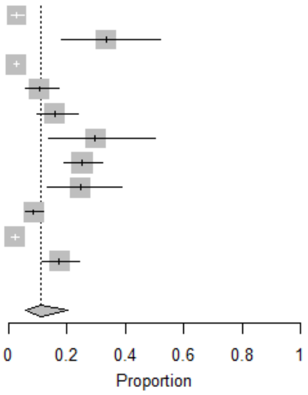

Gustatory dysfunction

Study	Events	Total	Proportion	95%-CI	Weight
Bellan	12	238	0.05	[0.03; 0.09]	12.7%
Cousyn	9	98	0.09	[0.04; 0.17]	11.7%
Daher	3	33	0.09	[0.02; 0.24]	7.8%
Gerhards	13	47	0.28	[0.16; 0.43]	12.2%
Jacobs	18	183	0.10	[0.06; 0.15]	13.5%
Leth	15	49	0.31	[0.18; 0.45]	12.4%
Otte	0	26	0.00	[0.00; 0.13]	2.3%
Peghin	62	599	0.10	[0.08; 0.13]	15.1%
Vaira	11	138	0.08	[0.04; 0.14]	12.4%
**Random effects model**		**1411**	**0.11**	**[0.07; 0.17]**	**100.0%**

Heterogeneity: $I^2 = 80\%$, $p < 0.01$

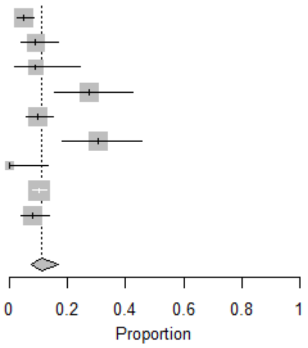

Headache

Study	Events	Total	Proportion	95%-CI	Weight
Bellan	0	238	0.00	[0.00; 0.02]	6.4%
Daher	5	33	0.15	[0.05; 0.32]	14.6%
Holmes	3	27	0.11	[0.02; 0.29]	13.2%
Jacobs	23	183	0.13	[0.08; 0.18]	16.8%
Leth	13	49	0.27	[0.15; 0.41]	16.1%
Mahmud	12	355	0.03	[0.02; 0.06]	16.3%
Peghin	16	599	0.03	[0.02; 0.04]	16.6%
**Random effects model**		**1484**	**0.07**	**[0.03; 0.16]**	**100.0%**

Heterogeneity: $I^2 = 91\%$, $p < 0.01$

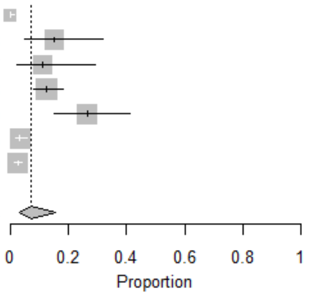

Diarrhoea

Study	Events	Total	Proportion	95%-CI	Weight
Bellan	3	238	0.01	[0.00; 0.04]	16.9%
Daher	3	33	0.09	[0.02; 0.24]	16.3%
Jacobs	7	183	0.04	[0.02; 0.08]	22.6%
Leth	4	49	0.08	[0.02; 0.20]	18.6%
Sonnweber	13	145	0.09	[0.05; 0.15]	25.6%
**Random effects model**		**648**	**0.05**	**[0.03; 0.10]**	**100.0%**

Heterogeneity: $I^2 = 68\%$, $p = 0.01$

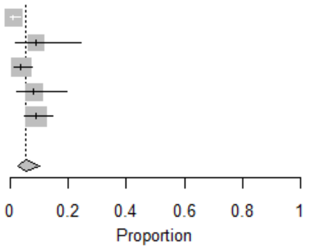

Chest pain

Study	Events	Total	Proportion	95%-CI	Weight
Bellan	1	238	0.00	[0.00; 0.02]	12.6%
Fernandez-de-Las-Penas	127	1950	0.07	[0.05; 0.08]	20.5%
Holmes	1	27	0.04	[0.00; 0.19]	12.4%
Leth	10	49	0.20	[0.10; 0.34]	19.1%
Mahmud	3	355	0.01	[0.00; 0.02]	17.0%
Peghin	5	599	0.01	[0.00; 0.02]	18.3%
**Random effects model**		**3218**	**0.03**	**[0.01; 0.08]**	**100.0%**

Heterogeneity: $I^2 = 91\%$, $p < 0.01$

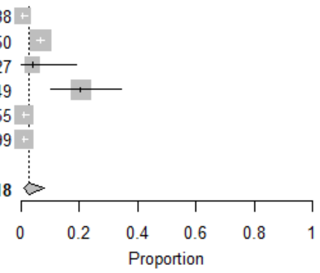

Table S3 Summarised results for meta-analysis by follow-up time subgroup

Symptom	Subgroup (weeks)	Number of studies	Number of patients	Pooled incidence (%) [95% CI]	I^2 (%)	P value
Fatigue	4-12	2	538	38 [28-51]	86	0.92
	> 12	7	2831	37 [18-62]	98	
Dyspnoea	4-12	3	571	20 [7-49]	96	0.99
	> 12	8	3248	21 [13-32]	96	
Olfactory dysfunction	4-12	6	1558	14 [5-35]	98	0.57
	> 12	7	1131	19 [11-31]	90	
Myalgia	4-12	3	571	7 [1-32]	93	0.38
	> 12	3	314	17 [5-47]	93	
Cough	4-12	3	571	20 [8-40]	94	0.18
	> 12	8	3248	9 [4-19]	95	
Gustatory dysfunction	4-12	4	452	9 [4-14]	0	0.36
	> 12	5	959	13 [6-26]	89	
Headache	4-12	3	571	9 [3-21]	88	0.60
	> 12	4	913	5 [1-24]	94	
Diarrhoea	4-12	2	216	5 [2-12]	39	0.91
	> 12	3	432	5 [2-14]	80	
Chest pain	4-12	1	355	1 [0-2]	NA	0.09
	> 12	5	2863	3 [1-10]	91	

Abbreviations: NA, not applicable.

Figure S2 Individual funnel plots for symptoms and signs included in the meta-analysis

<table>
<tr><th>Symptom</th><th>Funnel plot</th></tr>
<tr><td>Fatigue</td><td>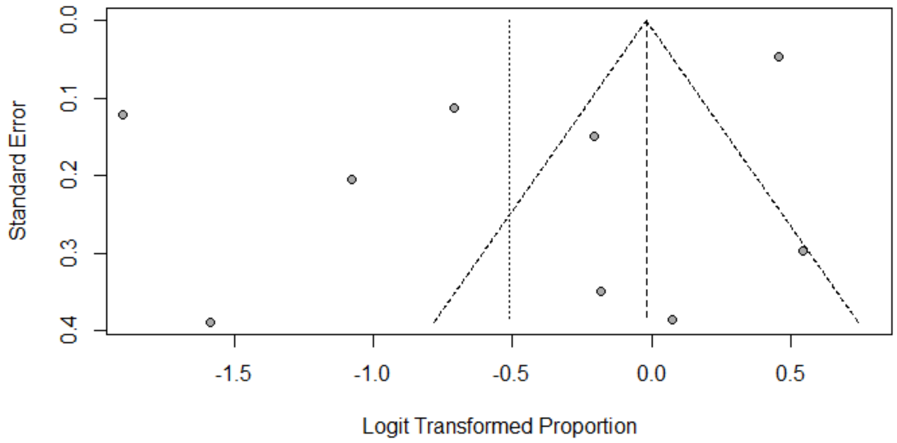
</td></tr>
<tr><td>Dyspnoea</td><td>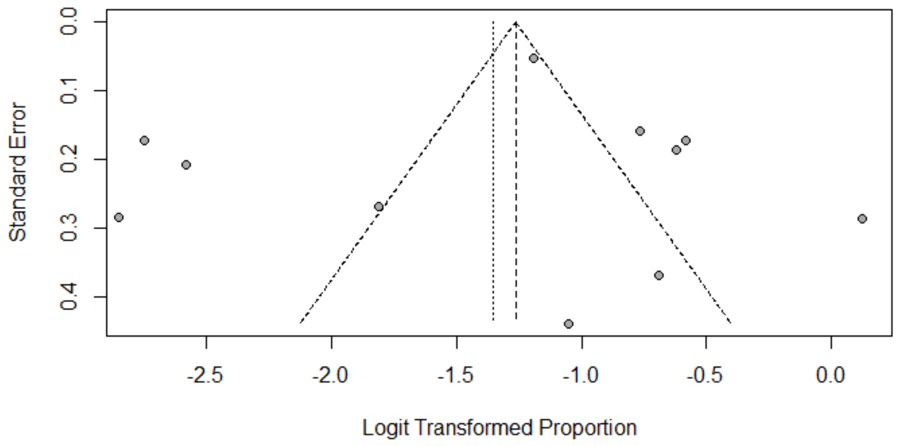
</td></tr>
<tr><td>Olfactory dysfunction</td><td>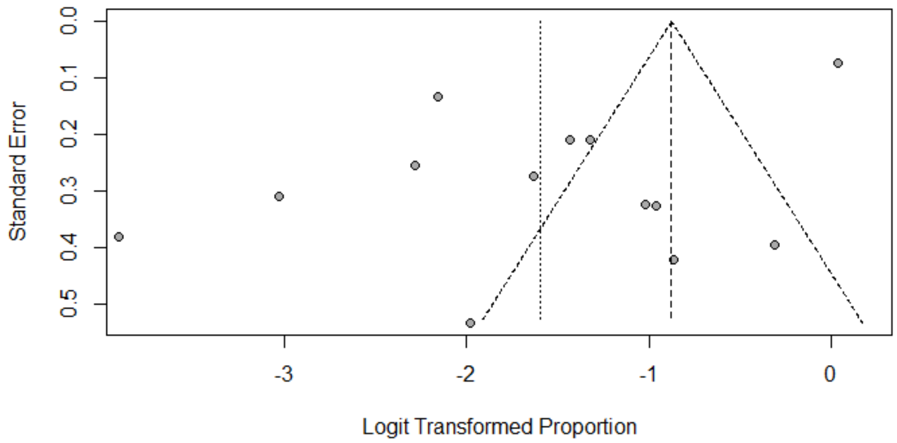
</td></tr>
</table>

Myalgia	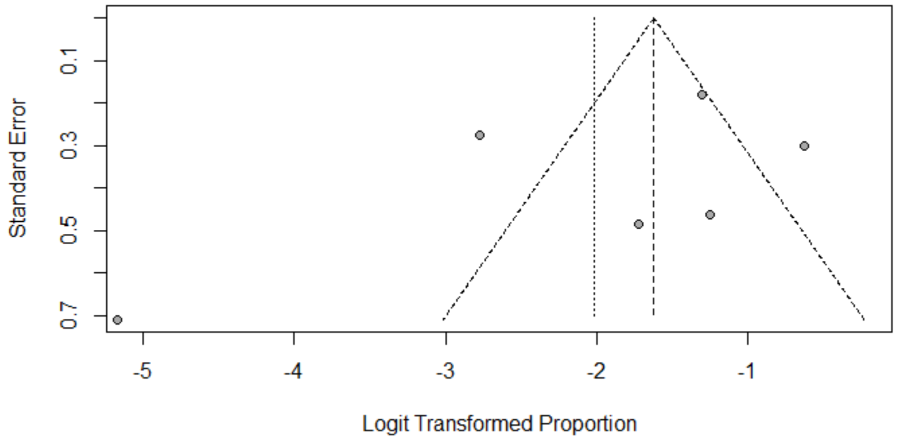
Cough	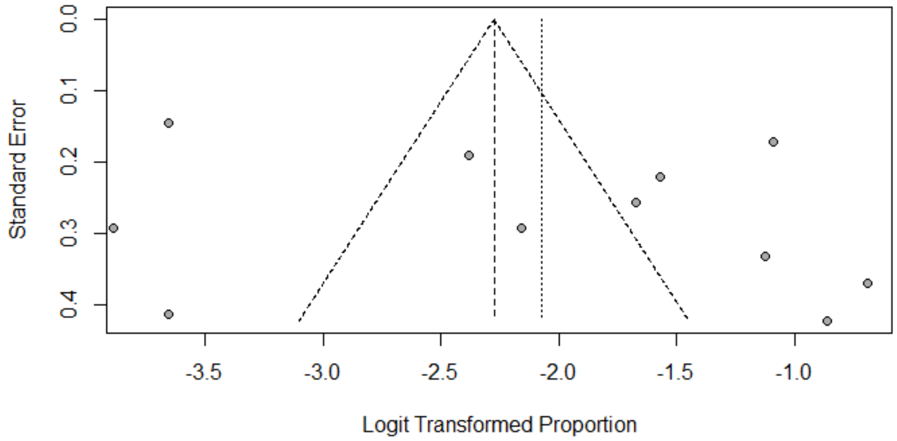
Gustatory dysfunction	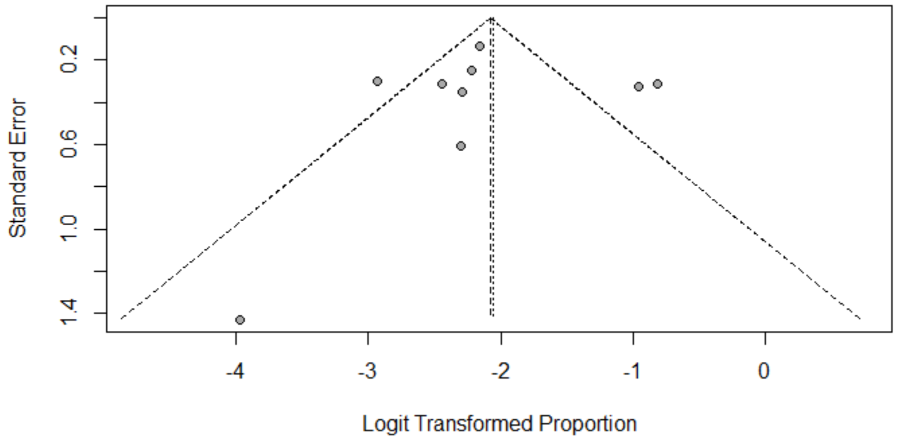

Headache	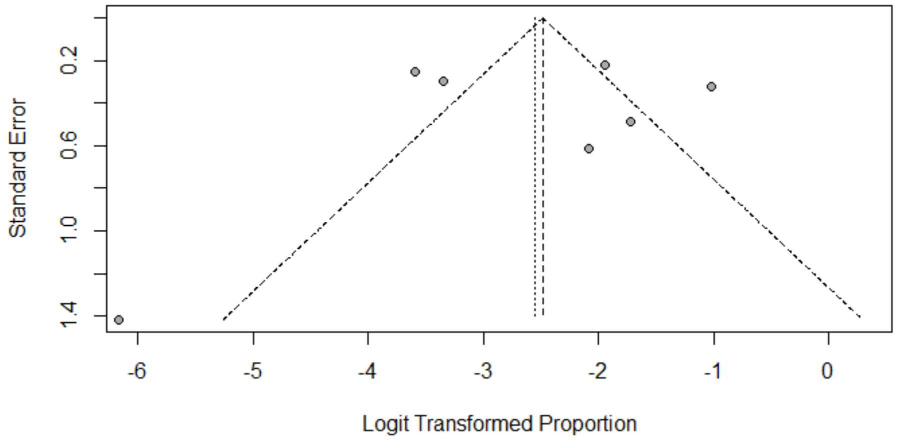
Diarrhoea	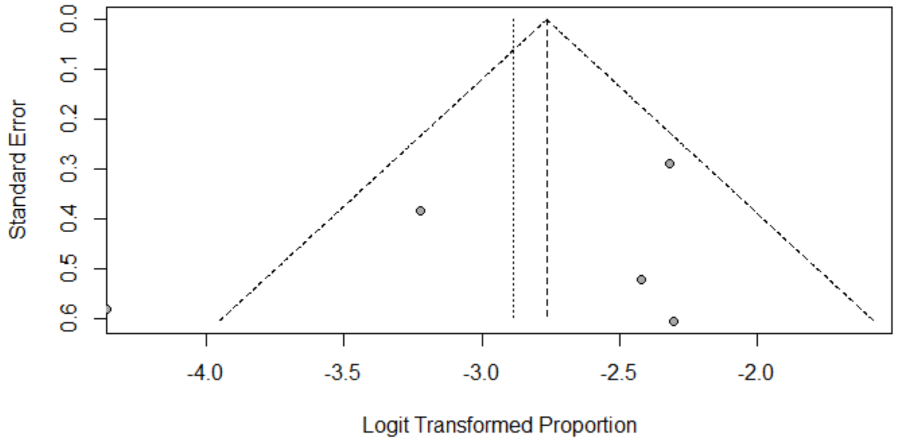
Chest pain	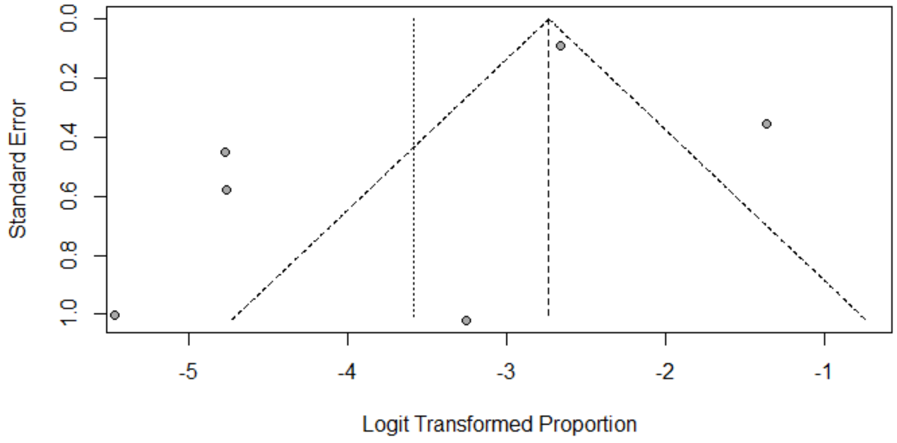
